# Clinical factors associated with early progression and grade 3–4 toxicity in patients with advanced non-small-cell lung cancers treated with nivolumab

**DOI:** 10.1371/journal.pone.0195945

**Published:** 2018-04-23

**Authors:** Coraline Dumenil, Marie-Ange Massiani, Jennifer Dumoulin, Violaine Giraud, Sylvie Labrune, Thierry Chinet, Etienne Giroux Leprieur

**Affiliations:** 1 Department of Respiratory Diseases and Thoracic Oncology, APHP—Ambroise Pare Hospital, Boulogne-Billancourt, France; 2 Department of Medical Oncology, René Huguenin Hospital, Curie Institute, Saint-Cloud, France; 3 EA4340, University of Versailles Saint-Quentin-en-Yvelines, Paris-Saclay University, Boulogne-Billancourt, France; Universidade do Algarve Departamento de Ciencias Biomedicas e Medicina, PORTUGAL

## Abstract

**Introduction:**

The prognosis of advanced non-small-cell lung cancer (NSCLC) has been improved by development of immune checkpoint inhibitors (ICIs) such as nivolumab for second-line treatment. As phase III trials include only selected patients, we here investigated the clinical factors associated with efficacy and safety of nivolumab in ‘real life’ patients with advanced NSCLC.

**Methods:**

Clinical and histological characteristics, therapies and survival data of all consecutive patients with advanced NSCLC included prospectively and treated by nivolumab in two French academic hospitals between February 2015 and December 2016 were examined.

**Results:**

Sixty-seven patients were included, mostly male (69%), current or former smokers (87%) with PS <2 (73%). Median age was 68.5 years and 42% were aged ≥70 years. According to uni- and multi-variate analyses, only PS 2 (OR = 0.17, 95% CI 0.03–0.99, *p* = 0.049) and number of previous treatment lines (OR = 0.33, 95% CI 0.13–0.85, *p* = 0.022) were significantly negatively associated with tumor control. Worse progression-free survival (PFS) was significantly associated with PS 2 (HR = 5.17, 95% CI 1.99–13.43, *p* = 0.001) and use of steroids (HR = 3.27, 95% CI 1.39–7.69, *p* = 0.006). Worse overall survival was associated with symptomatic brain metastasis (HR = 3.15, 95% CI 1.23–8.85, *p* = 0.029). Treatment-related adverse events occurred in 47 patients (70%), symptomatic brain metastasis being significantly associated with Grade ≥3 toxicity (OR = 8.13, 95% CI 1.21–55.56, *p* = 0.031). Age and nutritional status were not associated with response, PFS, OS or toxicity.

**Conclusion:**

Our results suggest that nivolumab is not beneficial or safe for patients with PS 2 and symptomatic brain metastases.

## Introduction

Non-small cell lung cancer (NSCLC) is the leading cause of cancer related–death worldwide [1]. The treatment of NSCLC has recently been improved by development of immune checkpoint inhibitors (ICIs), which target inhibitor pathways such as programmed death 1 (PD1)/programmed death-ligands 1 (PDL1), thus blocking the immune responses of tumor-infiltrating lymphocytes, allowing proliferation of tumor cells [2]. Nivolumab, a fully human IgG4 PD-1 ICI antibody, was the first agent to show efficacy in second-line treatment of patients with NSCLC in two recent phase III trials and had a favorable safety profile compared with docetaxel [3, 4]. A new era of immunotherapy has begun with encouraging results from randomized phase II and III trials, putting into question and extending the potential indications for ICIs for NSCLC [5]. However, patients included in these trials all had performance status (PS) 0–1, did not have active or untreated brain metastases, and were relatively young (only 7% and 11% of ≥75 years-old patients in Checkmate-057 and Checkmate-017 trials, respectively). Thus, benefit and safety of ICIs for all patients with advanced NSCLC in ‘real life’ is still unanswered. Seventeen percent of patients with Stage IV NSCLC have central nervous system (CNS) metastases at initiation of first-line treatment [6] and around one third of patients with NSCLC have a PS of 2 [7].

The objectives of this study were to determine the clinical factors associated with the efficacy and safety of nivolumab in ‘real-life’ patients with advanced NSCLC, including elderly patients and those with poor PS, brain metastases, or poor nutritional status at the time of nivolumab.

## Materials and methods

### Patients

Records of all consecutive patients with advanced NSCLC treated by nivolumab (3 mg/ kg every two weeks) between February 2015 and December 2016 were retrieved from a database of prospectively collected information on all patients with NSCLC treated in two French academic hospitals (Ambroise Paré Hospital and René Huguenin Hospital). The following clinical data were collected from medical charts: age at diagnosis; sex; ethnicity; smoking history; tumor histologic type according to the 2004 World Health Organization Criteria ^8^; Stage IIIB or IV according to the International Association for the Study of Lung Cancer Lung Cancer Staging Project [8]; locations of metastatic sites; mutation status (*EGFR/ KRAS/ HER2/ BRAF* mutations or *ALK* translocation); PS, use of oral or intravenous corticosteroids (CS), and body mass index (BMI) at the time of nivolumab initiation; loss of weight (< or ≥ 5% since diagnosis); and serum albumin concentration during the month prior to first administration of nivolumab. Smoking status was defined as never smoker (<100 cigarettes during lifetime), former smoker (quit > 1 year prior to diagnosis), or current smoker (still smoking or quit ≤1 year prior to diagnosis). All patients were evaluated with a CT scan of the chest and upper abdomen every four cycles of nivolumab.

Previous treatment(s) before nivolumab (surgery, radiotherapy or chemotherapy), progression-free survival (PFS), overall survival (OS), best response to nivolumab, and time to progression before nivolumab (delay between the last dose of previous treatment and the administration of the first cycle of nivolumab) were also recorded.

Response criteria were defined according to the guidelines of Response Evaluation Criteria in Solid Tumors (RECIST, version 1.1) [9]. Adverse events of nivolumab therapy were graded according to the National Cancer Institute Common Terminology Criteria for Adverse Events (NCI CTCAE, version 4.0).

### Ethics

The retrieval of data from our prospectively collected database conformed to the rules of the Commission Nationale de l’Informatique et des Libertés. This study was approved by the Institutional Review Board of the French Learned Society for Respiratory Medicine (Société de Pneumologie de Langue Française). The data were fully anonymized before the authors accessed them. No written consent was mandatory for this retrospective analysis, as approved by the Institutional Review Board of the French Learned Society for Respiratory Medicine.

### Statistical analyses

Clinical, biological, and pathological data were compared between patients with controlled (including complete response, partial response and stable disease) versus progressive disease and with versus without nivolumab-related Grade 3–4 toxicities. Associations with qualitative variables were assessed using the χ^2^ whereas comparisons of continuous variables were assessed using the Mann–Whitney test (univariate analyses). Continuous variables are expressed as mean (± standard deviation) if normally distributed or median (interquartile range, IQR) if non-normally distributed. Independent variables with *p* < 0.15 in the univariate analyses were subjected to multivariate analysis. Multivariate analyses for tumor control and toxicity were performed using a logistic regression model and those for PFS and OS using a Cox model. PFS and OS were estimated using the Kaplan–Meier method (*p*-value calculated using log-rank test). The censoring date was 27/12/2016 for PFS and 15/11/2017 for OS. Results were considered significant if the *p*-value was < 0.05. Statistical tests were performed using Xlstat 2017 software (Addinsoft).

## Results

### Patient characteristics

We analyzed data of a 67 patients (48 and 19 patients from Ambroise Paré Hospital and René Huguenin Hospital, respectively): their characteristics are summarized in Table 1. Median age at diagnosis was 68.5 years (IQR 60–77), 42% being aged ≥ 70 years). Most patients were male (69%), Caucasian (97%), in fair or good physical condition (PS < 2, 73%), not malnourished (BMI ≥ 18.5 kg/m^2^ and serum albumin ≥ 30 g/L in 87% and 69%, respectively). Most patients had a history of smoking (43% current and 43% former smokers) with a median consumption of 35 pack-years. Adenocarcinoma was the most frequent histological type (70%). The tumors were mostly wild-type (67%) and Stage IV (82%). Metastases at diagnosis were mainly to lungs (40%), bones (26%), adrenal glands (22%), and CNS (19%). Prior to first cycle of nivolumab, ten patients had symptomatic brain metastasis (15%), including seven already treated by whole brain or stereotaxic radiotherapy (10%) (five at least one month before first cycle of nivolumab [7%] and two during the previous month [3%]) (Table 2). Ten patients (15%) received systemic CS at the time of first administration of nivolumab: five for symptomatic CNS metastasis with symptoms of intracranial hypertension (8%), two for hypercalcemia (3%), one for medullary compression (1%), one for concomitant glioma (1%) and one for spinal cord compression (1%).

**Table 1:** Demographic, Pathologic, and Molecular Characteristics of the patients.

	All patients n = 67	Controlled disease (n = 32)	Progressive disease (n = 35)	*p*-value*
**Age (years)**				
<70	39 (58)	18 (27)	21 (31)	0.756
≥70	28 (42)	14 (21)	14 (21)	
**Gender**				
Male	46 (69)	23 (34)	23 (34)	0.587
Female	21 (31)	9 (14)	12 (18)	
**Smoking history**				
Current smokers	29 (43)	14 (21)	15 (22)	0.224
Former smokers	29 (43)	16 (24)	13 (19)	
Never smokers	9 (14)	2 (3)	7 (11)	
**Histological subtypes**				
Adenocarcinoma	47 (70)	25 (37)	22 (33)	0.366
Squamous-cell carcinoma	17 (25)	6 (9)	11 (16)	
Others	3 (5)	1 (2)	2 (3)	
**Mutation status**				0.064
*EGFR*	0	0	0	
*ALK*	0	0	0	
*Kras*	19 (28)	13 (30)	6 (9)	
*Braf*	1 (1)	1 (1)	0	
*HER2*	2 (3)	0	2 (3)	
WT	45 (67)	18 (27)	27 (40)	
**Stage**				
IIIB	12 (18)	4 (6)	6 (9)	0.594
IV	55 (82)	28 (42)	29 (43)	
**Number of metastatic sites**				
0	10 (15)	4 (6)	6 (9)	0.270
1	35 (52)	20 (30)	15 (22)	
>1	22 (33)	8 (12)	14 (21)	
**CNS metastasis at diagnosis**				
Yes	11 (16)	3 (5)	8 (12)	0.137
No	56 (84)	29 (43)	27 (40)	
**Symptomatical CNS metastasis at 1st cycle of nivolumab**				
Yes	10 (15)	1 (2)	9 (13)	0.010
No	57 (85)	31 (46)	26 (39)	
**Systemic steroid use at 1st cycle of nivolumab**				
Yes	10 (15)	1 (2)	9 (13)	0.031
No	57 (85)	31 (46)	26 (39)	
**Number of lines of chemotherapy before nivolumab**	1 (1–2)	1 (1–1)	1 (1–2.5)	0.017
**Best response to chemotherapy before nivolumab**				
Partial response	16 (24)	9 (14)	7 (11)	0.547
Stable disease	28 (43)	11 (17)	17 (26)	
Progression disease	21 (32)	10 (15)	11 (17)	
**Time to progression before nivolumab (days)**	30 (23–90)	60 (25–165)	30 (21–60)	
**PS at 1st cycle of nivolumab**				
<2	49 (73)	28 (42)	21 (31)	0.026
2	18 (27)	4 (6)	14 (21)	
**BMI at 1st cycle of nivolumab (kg/m**^**2**^**)**				0.011
<18.5	9 (13)	0 (0)	9 (13)	
≥18.5	58 (87)	29 (43)	29 (43)	
Serum albumin level at 1st cycle of nivolumab (g/L)**				0.005
<30	20 (31)	6 (9)	14 (22)	
≥30	45 (69)	26 (40)	19 (29)	
**Loss of weight compared to diagnosis**				0.039
<5%	50 (75)	24 (36)	26 (39)	
≥5%	17 (25)	8 (12)	9 (13)	

Data presented as median (interquartile range) or n (%). Abbreviations: PS: performans status WT: wild-type; CNS: central nervous system; 1st: first; BMI: body mass index.

*p-value from Mann–Whitney, chi-squared, or Fisher's exact test as required

** two missing data

**Table 2:** Demographic, Pathologic, and Molecular Characteristics of the 10 patients with symptomatic CNS metastasis prior 1^st^ administration of nivolumab.

Pt	PS	Nb of CNS mets	Neurological symptoms	Dose of CS (mg/d)	Previous brain RT and delay before nivolumab	Lx and number of cycles of nivolumab	Best response to nivolumab	TTP (months)	Grade 3–4 toxicity	Further anti-tumor treatment
#1	1	3	ICH	40	WBR, 2 months	L4, 4 cycles	PD	1.77	0	yes
#2	2	>3	ICH	30	none	L2, 2 cycles	PD	0.93	fatigue, anorexia, worsening of ICH	no
#3	1	>3	ICH	20	WBR, 3 months	L2, 3 cycles	PD	1.67	fatigue, anorexia	no
#4	1	1	headache	0	stereotaxic RT, 8 days	L2, 5 cycles	PD	1.77	0	yes
#5	1	3	ICH	10	WBR, 2 months	L2, 12 cycles	SD	NA	0	NA
#6	0	>3	psychomotor slowdown	0	WBR, 9 months	L3, 2 cycles	PD	1.47	fatigue, anorexia, worsening of ICH	no
#7	2	>3	confusion, psychomotor slowdown	0	WBR, 3 weeks	L2, 5 cycles	PD	3.73	fatigue, anorexia	no
#8	0	1	confusion	0	none	L4, 3 cycles	PD	1.17	fatigue, anorexia, worsening of ICH	no
#9	1	>3	ICH	30	WBR, 16 months	L5, 1 cycle	PD	0.23	worsening of ICH	no
#10	2	2	dysarthria, facial paralysis	0	none	L2, 2 cycles	PD	0.5	fatigue, anorexia, worsening of ICH	no

Abbreviations: Pt: patient; Nb: number; CNS: central nervous system; mets: metastases; PS: performance status; CS: corticosteroids; mg: milligram; RT: radiotherapy; WBR: Whole brain radiotherapy; ICH: intracranial hypertension; Lx: line of treatment; PD: progressive disease; SD: stable disease; NA: not available.

### Efficacy of nivolumab

Sixty-six patients (99%) received nivolumab as ≥ second-line treatment. One patient with PS 2 received nivolumab as first-line treatment for metastatic disease after having received adjuvant chemotherapy. The median number of nivolumab perfusions per patient was five (IQR 3–12). The disease control rate was 48% (n = 32) (34% and 13% stable disease and partial response, respectively), whereas 52% patients exhibited tumor progression (n = 35).

Regarding tumor control, univariate analysis showed that PS 2 at the time of nivolumab initiation (21% vs. 6%, *p* = 0.026), number of previous treatment lines (1 [1–2.5] vs. 1 [1–1], *p* = 0.017), presence of symptomatic brain metastases (13% vs. 2%, *p* = 0.010), use of systemic CS before first cycle of nivolumab (13% vs. 2%, *p* = 0.031), poor nutritional status with BMI < 18.5 kg/ m^2^ (13% vs. none, *p* = 0.011), albumin concentration < 30g/L (22% vs. 9%, *p* = 0.005) and ≥5% loss of weight (13% vs. 12%, *p* = 0.039) were significantly associated with progressive disease on nivolumab (Table 1). Multivariate analysis showed that PS 2 (OR = 0.17, 95% CI 0.03–0.99, *p* = 0.049) and number of previous treatment lines (OR = 0.33, 0.13–0.85, *p* = 0.022) were independently associated with progressive disease (Table 3). No significant differences were identified for age, sex, smoking history, histological subtypes, mutation status, TNM stage, and multisite metastases.

**Table 3:** Multivariate analysis of disease control as best tumor response with nivolumab.

	Multivariate analysis OR CI [95%]	*p*-value^¥^
*Kras* mutation	2.07 [0.45–9.52]	0.349
*HER2* mutation	0.14 [0.001–15.78]	0.412
CNS metastatis at diagnosis (yes vs. no)	0.50 [0.07–3.70]	0.493
Symptomatic CNS metastasis (yes vs. no)	0.38 [0.05–3.27]	0.380
Systemic steroid use at 1st cycle of nivolumab	0.39 [0.04–3.69]	0.410
Number of lines before nivolumab	0.33 [0.13–0.85]	0.022
Time to progression before nivolumab (days)	1.01 [0.99–1.01]	0.161
PS = 2 at 1st cycle of nivolumab	0.17 [0.03–0.99]	0.049
BMI at 1st cycle of nivolumab <18.5 kg/m^2^	1.59 [0.17–14.9]	0.692
Albumin level at 1st cycle of nivolumab < 30 g/L	0.85 [0.15–4.86]	0.852

Abbreviations: OR: odds ratio; PS: performans status; CNS: central nervous system; 1st: first; BMI: body mass index.

^¥^Logistic regression model; independent variables with p < 0.15 in univariate analyses were included in the multivariate analyses.

**Fig 1:**
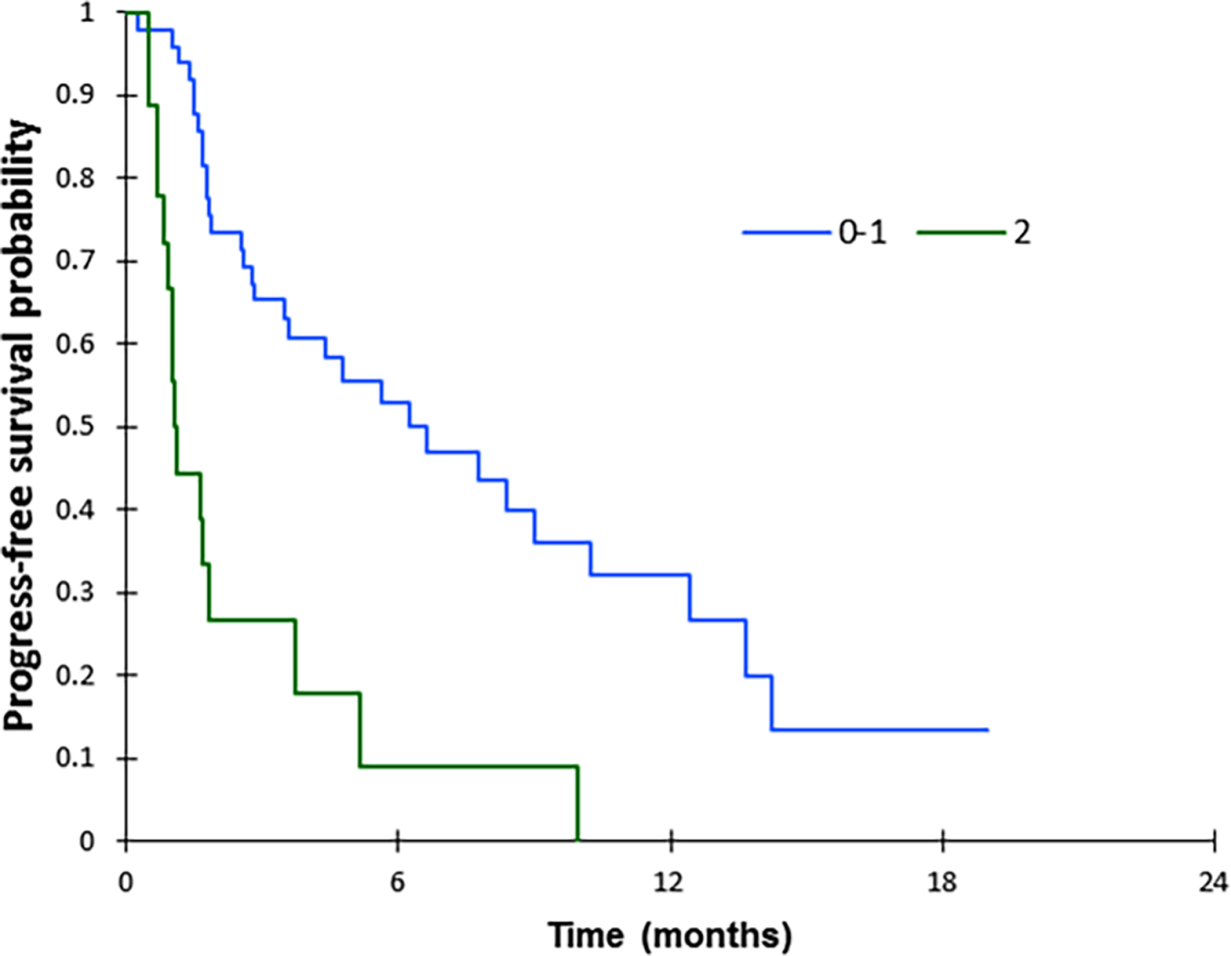
Progression-free survival according to performans status before first cycle of nivolumab. p<0.001 (log-rank test).

**Fig 2:**
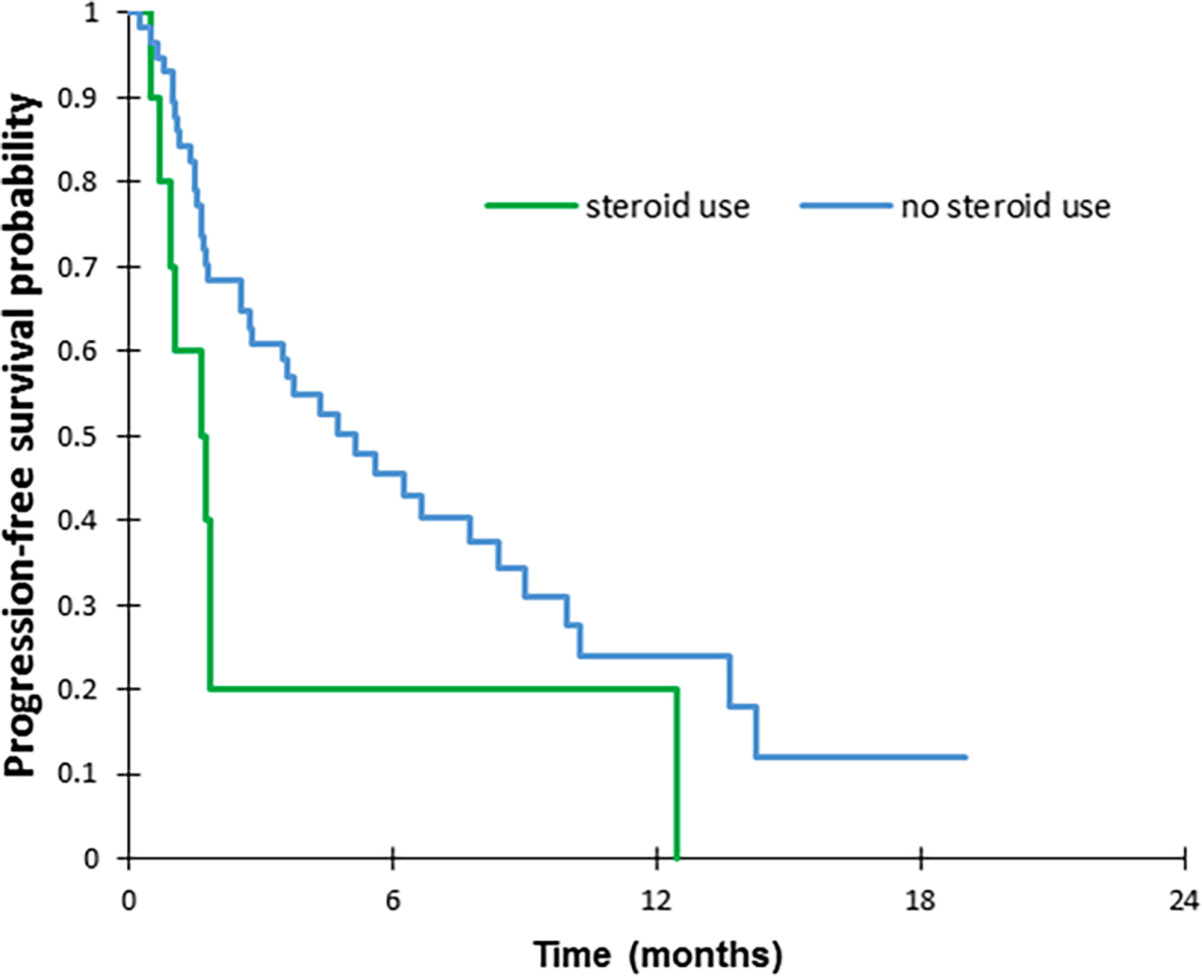
Progression-free survival according to steroid use before first cycle of nivolumab. p = 0.027 (log-rank test).

**Fig 3:**
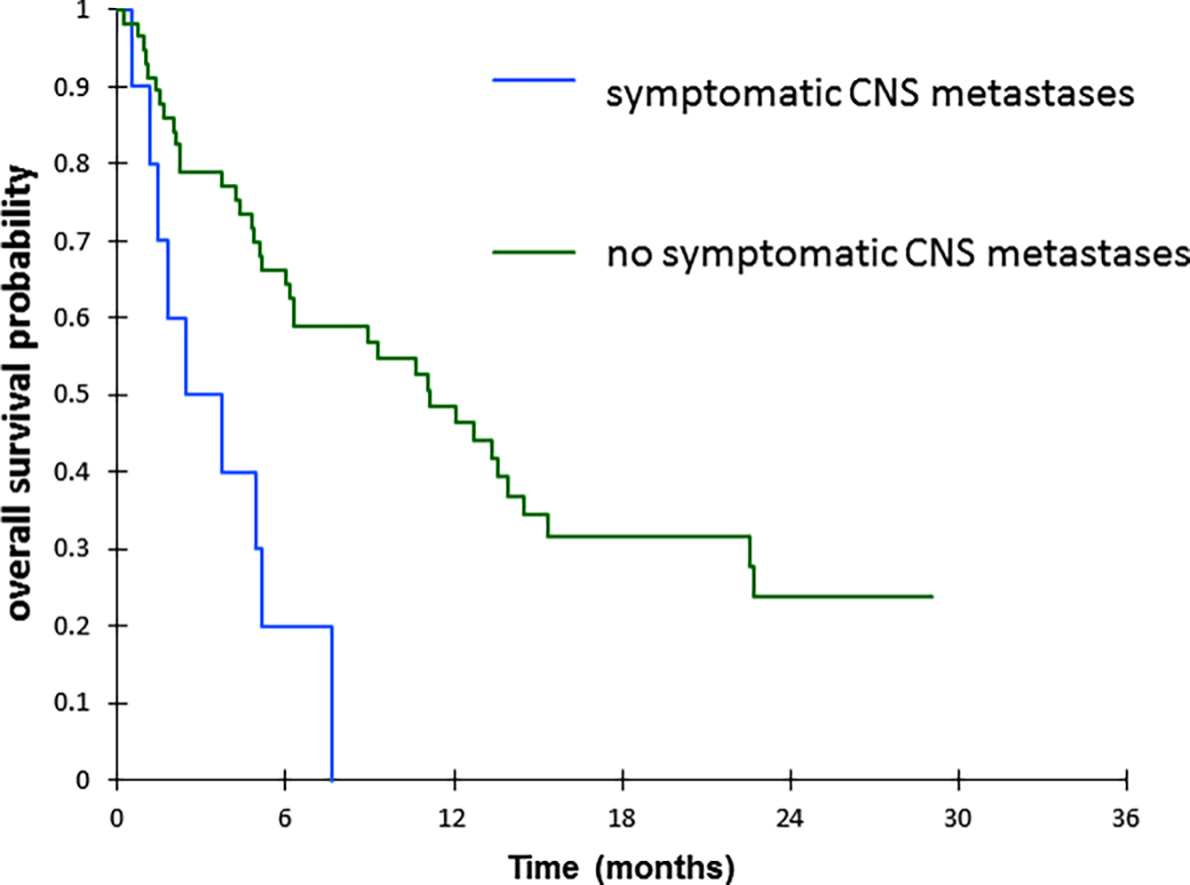
Overall survival according to the presence/absence of symptomatic central nervous system (CNS) metastases. p = 0.001 (log-rank test).

Median PFS and OS on nivolumab were 3 months (IQR 1.6–6.6) and 6.3 months (IQR 3.1–13.5), respectively. Ten patients (15%) were receiving CS before the first cycle of nivolumab (median dose 25 mg, IQR 12.5–37.5). According to univariate analysis, lower PFS was significantly associated with PS 2 (HR = 4.01, 95% CI 2.12–7.60, *p*<0.0001) (Table 4) with a median PFS of 1.1 months (IQR 0.8–3.7) vs. 6.6 months (IQR 1.9–13.7) for PS < 2 (*p* < 0.0001) (Fig 1) and with CS administrated at the time of the first cycle of nivolumab (HR = 2.25, 95% CI 1.08–4.70, *p* = 0.031) with a median PFS of 1.7 months (IQR 0.9–1.9) vs. 5.1 months (IQR 1.70–10.2) for patients without CS (*p* = 0.027) (Fig 2). Multivariate analysis confirmed that PS 2 and use of CS were independent predictors for a shorter PFS (HR = 5.17, 95% CI 1.99–13.43, *p* = 0.001 and HR = 3.27, 95% CI 1.39–7.69, *p* = 0.006, respectively). No significant differences were noted for clinical, biological or nutrition data, histological subtype, TNM stage, or previous response to other chemotherapy. For OS, according to univariate analyses, lower OS was significantly associated with non-adenocarcinoma and non-squamous histology (HR = 3.70, 95%CI 1.07–12.74, *p* = 0.038), the presence of symptomatic CNS metastases (HR = 3.61, 95% CI 1.64–7.94, *p* = 0.001), with CS administrated at the time of the first cycle of nivolumab (HR = 2.54, 95% CI 1.21–5.32, p = 0.014), with PS 2 (HR = 2.42, 95% CI 1.32–4.47, *p* = 0.005) and Albumine level at 1^st^ cycle of nivolumab <30g/l (HR = 2.54, 95% CI 1.38–4.69, *p* = 0.003) (Table 5). Multivariate analyses showed that only the presence of symptomatic CNS metastases was an independent predictor for a shorter OS (HR = 3.15, 95% CI 1.23–8.85, p = 0.029). Median OS was 3.1months in case of symptomatic brain metastases, versus 11.2 months for other patients (*p* = 0.001) (Fig 3). No significant differences were noted for other parameters.

**Table 4:** Factors associated with progression-free survival.

	Univariate analysis		Multivariate analysis	
	HR [CI95%]	*p-value*^¥^* *	HR [CI95%]	*p-value*^¥^* *
**Age (<70 vs. ≥70 years)**	1.01 [0.98–1.05]	0.539		
**Gender (male vs. female)**	0.85 [0.45–1.61]	0.609		
**Smoking history**				
Current smokers	1			
Former smokers	0.85 [0.45–1.58]	0.549		
Never smokers	1.93 [0.79–4.68]	0.148	2.67 [0.87–8.14]	0.086
**Histological subtypes**				
Adenocarcinoma	1			
Squamous-cell carcinoma	1.46 [0.81–2.62]	0.211		
Others	2.31 [0.53–10.12]	0.266		
**Mutational status**				
*Kras*	0.53 [0.26–1.07]	0.076	0.66 [0.30–1.46]	0.305
*Braf*	2.71 [0.34–21.54]	0.346		
*HER2*	8.99 [1.82–44.40]	0.007	2.85 [0.41–19.67]	0.288
WT	1			
**Stage (IIIb vs. IV)**	0.83 [0.37–1.87]	0.651		
**Number of metastatic sites**				
0	1			
1	0.69 [0.29–1.62]	0.391		
>1	1.13 [0.47–2.73]	0.793		
**CNS metastasis at diagnosis (yes vs. no)**	0.79 [0.38–1.68]	0.542		
**Symptomatic CNS metastasis at 1**^**st**^ **cycle of nivolumab (yes vs. no)**	1.48 [0.71–3.10]	0.292		
**Systemic steroid use at 1st cycle of nivolumab (yes vs. no)**	2.25 [1.08–4.70]	0.031	3.27 [1.39–7.69]	0.006
Number of lines before nivolumab*	1.17 [0.88–1.55]	0.272		
**Best response to chemotherapy before nivolumab**				
Partial response	1			
Stable disease	0.70 [0.31–1.60]	0.963		
Progression disease	1.14 [0.25–5.23]	0.868		
Time to progression before nivolumab*	0.99 [0.99–0.99]	0.018	0.99 [0.99–1.00]	0.057
**PS = 2 at 1st cycle of nivolumab (yes vs. no** **)**	4.01 [2.12–7.60]	<0.0001	5.17 [1.99–13.43]	0.001
**BMI at 1st cycle of nivolumab (<18.5 vs. ≥18.5 kg/ m**^**2**^**)**	1.07 [0.42–2.73]	0.888		
**Albumin serum level at 1st cycle of nivolumab (<30 vs. ≥30 g/ L)**	2.26 [1.24–4.12]	0.008	0.82 [0.33–2.03]	0.669
**Loss of weight compared to diagnosis (<5 vs. ≥5%)**	1.39 [0.73–2.65]	0.313		

Data presented as median (interquartile range) or n (%). Abbreviations: HR: hazard ratio; WT: wild-type; PS: performans status; CNS: central nervous system; 1st: first; BMI: body mass index.

*continuous variables.

^¥^p-value from Cox model in the univariate and multivariate analysis. Independent variables with p<0.15 in univariate analyses were included in the multivariate analyses.

**Table 5:** Factors associated with overall survival.

	Univariate analysis		Multivariate analysis	
	HR [CI95%]	*p-value*^¥^* *	HR [CI95%]	*p-value*^¥^* *
**Age (<70 vs. ≥70 years)**	0.22 [0.81–2.59]	0.215		
**Gender (male vs. female)**	1.25 [0.68–2.31]	0.475		
**Smoking history**				
Current smokers	1		1	
Former smokers	1.09 [0.58–2.05]	0.789	1.07 [0.52–2.20]	0.857
Never smokers	2.15 [0.89–5.22]	0.089	2.30 [0.65–8.14]	0.196
**Histological subtypes**				
Adenocarcinoma	1		1	
Squamous-cell carcinoma	1.46 [0.80–2.66]	0.220	1.38 [0.62–3.12]	0.432
Others	3.70 [1.07–12.74]	0.038	2.88 [0.74–11.19]	0.126
**Mutational status**				
*Kras*	0.50 [0.25–1.02]	0.056	0.70 [0.30–1.64]	0.415
*Braf*	3.21 [0.40–25.59]	0.272	4.40 [0.43–45.06]	0.212
*HER2*	4.10 [0.88–19.03]	0.072	2.85 [0.40–20.30]	0.295
WT	1		1	
**Stage (IIIb vs. IV)**	0.78 [0.35–1.75]	0.545		
**Number of metastatic sites**				
0	1			
1	0.61 [0.26–1.43]	0.251		
>1	1.20 [0.50–2.90]	0.690		
**CNS metastasis at diagnosis (yes vs. no)**	0.96 [0.43–2.16]	0.924		
**Symptomatic CNS metastasis at 1**^**st**^ **cycle of nivolumab (yes vs. no)**	3.61 [1.64–7.94]	0.001	3.15 [1.23–8.85]	0.029
**Systemic steroid use at 1st cycle of nivolumab (yes vs. no)**	2.54 [1.21–5.32]	0.014	1.31 [0.51–3.38]	0.579
Number of lines before nivolumab*	1.20 [0.92–1.56]	0.190		
**Best response to chemotherapy before nivolumab**				
Partial response	1			
Stable disease	0.74 [0.35–1.56]	0.427		
Progression disease	0.67 [0.30–1.51]	0.336		
Time to progression before nivolumab*	0.99 [0.99–1.00]	0.098	0.99 [0.99–1.00]	0.536
**PS = 2 at 1st cycle of nivolumab (yes vs. no** **)**	2.42 [1.32–4.47]	0.005	2.20 [0.89–5.42]	0.086
**BMI at 1st cycle of nivolumab (<18.5 vs. ≥18.5 kg/ m**^**2**^**)**	1.29 [0.55–3.05]	0.561		
**Albumin serum level at 1st cycle of nivolumab (<30 vs. ≥30 g/ L)**	2.54 [1.38–4.69]	0.003	1.31 [0.53–3.21]	0.559
**Loss of weight compared to diagnosis (<5 vs. ≥5%)**	1.09 [0.56–2.11]	0.799		

Data presented as median (interquartile range) or n (%). Abbreviations: HR: hazard ratio; WT: wild-type; PS: performans status; CNS: central nervous system; 1st: first; BMI: body mass index.

*continuous variables.

^¥^p-value from Cox model in the univariate and multivariate analysis. Independent variables with p<0.15 in univariate analyses were included in the multivariate analyses.

### Nivolumab toxicity

Treatment-related adverse events (AEs) occurred in 47 patients (70%). The commonest AEs of any grade were fatigue (n = 40, 85%), anorexia (n = 31, 66%), and fever (n = 10, 21%) (S1 Table). Severe AEs (Grade ≥3) occurred in 28 patients (42%) and led to treatment discontinuation in 21 patients (31%). No toxicity-related deaths occurred. The most frequent Grade 3–4 events that led to interruption of nivolumab were fatigue and anorexia (n = 16, 24%) and exacerbation of neurologic symptoms (n = 9, 13%) such as consciousness disorders, nausea and headache with intracranial hypertension. Six of these nine patients had symptomatic CNS metastases before beginning nivolumab.

According to univariate analysis, Grade ≥3 AEs were significantly associated with symptomatic CNS metastases (12% vs. 3%, *p* = 0.008), PS 2 (19% vs. 9%, *p* = 0.012) and serum albumin at the time of the first cycle of nivolumab <30 g/L (20% vs. 10%, *p* = 0.012) (Table 6). Only symptomatic CNS metastases were significantly associated with worse toxicity (OR = 8.13, 95% CI 1.21–55.56, *p* = 0.031) according to multivariate analysis. Clinical data (age, sex, smoking history, PS, malnutrition), histological type, Stage, and previously administered treatment were not associated with a higher risk of toxicity of nivolumab.

**Table 6:** Univariate and multivariate analysis of the risk of occurrence of grade 3–4 toxicity with nivolumab (n = 67).

	Univariate analysis			Multivariate analysis		
	Grade 3–4	No grade 3–4	*p-value*^¥^	HR [CI95%]		*p-value*^¥^
**Age (years)**						
<70	16 (24)	23 (34)	0.881			
≥70	12 (18)	16 (24)				
**Gender**						
Male	22 (33)	24 (36)	0.138	1		
Female	6 (9)	15 (22)		0.27	0.06–1.19	0.085
**Smoking history**						
Current smokers	10 (15)	19 (28)	0.559			
Former smokers	14 (21)	15 (22)				
Never smokers	4 (6)	5 (8)				
**Histological subtypes**						
Adenocarcinoma	13 (19)	34 (51)	0.001	1		
Squamous-cell carcinoma	12 (18)	5 (8)		2.51	0.71–8.94	0.154
Others	3 (4)	0		18.56	0.32–1086.23	0.160
**Mutation status**						
*EGFR*	0	0	0.213			
*ALK*	0	0				
*Kras*	5 (7)	16 (23)				
*Braf*	0	1 (1)				
*HER2*	1 (1)	1 (1)				
WT	22 (33)	23 (34)				
**Stage**						
IIIB	3 (4)	7 (11)	0.412			
IV	25 (37)	32 (48)				
**Number of metastatic sites**						
0	3 (4)	7 (11)	0.129	1		
1	12 (18)	23 (34)		0.39	0.07–2.39	0.310
>1	13 (19)	9 (14)		1.62	0.25–10.72	0.617
**CNS metastasis at diagnosis**						
Yes	6 (9)	5 (7)	0.348			
No	22 (33)	34 (51)				
**Symptomatic CNS metastasis at 1**^**st**^ **cycle of nivolumab**						
Yes	8 (12)	2 (3)	0.008	8.13	1.21–55.56	0.031
No	20 (30)	37 (55)		1		
**Systemic steroid use at 1st cycle of nivolumab**						
Yes	6 (9)	5 (7)	0.348			
No	22 (33)	34 (51)				
**Number of lines of chemotherapy before nivolumab**	1 (1–2)	1 (1–2)	0.290			
Best response to chemotherapy before nivolumab*						
Partial response	8 (12)	8 (12)	0.333			
Stable disease	13 (20)	15 (23)				
Progression disease	6 (9)	15 (23)				
**Time to progression before nivolumab (days)**	42 (28–105)	30 (21–90)	0.590			
**PS at 1st cycle of nivolumab**						
<2	16 (24)	33 (48)	0.012	1		
2	12 (19)	6 (9)		3.32	0.66–16.59	0.144
**BMI at 1st cycle of nivolumab (kg/m**^**2**^**)**						
<18.5	2 (3)	7 (10)	0.201			
≥18.5	26 (39)	32 (48)				
Serum albumin level at 1st cycle of nivolumab (g/L)**						
<30	13 (20)	7 (10)	0.012	1.17	0.25–4.93	0.884
≥30	15(22)	32 (4)		1		
**Loss of weight compared to diagnosis**						
<5%	21 (31)	29 (43)	0.953			
≥5%	7 (10)	10 (15)				

Data presented as median (interquartile range) or n (%). Abbreviations: OR: odds ratio; WT: wild-type; PS: performans status; CNS: central nervous system; 1^st^: first; BMI: body mass index.

^**¥**^ *p*-value from Mann–Whitney or chi-squared in the univariate analyses. Logistic regression model; independent variables with *p* < 0.15 in univariate analyses were included in the multivariate analyses.

*One patient without previous line of chemotherapy and one missing data

**Two missing data.

## Discussion

In this study, we analyzed the efficacy and toxicity of nivolumab in 67 patients with advanced NSCLC to determine clinical factors associated with early progressive disease or high grade toxicity. We found that progressive disease and lower PFS were significantly more frequent in patients with PS 2 at the time of nivolumab initiation. CNS metastases were associated with worse OS and adverse events with nivolumab, and CS before the first cycle of nivolumab had also a negative impact on PFS. Importantly, in our cohort, we found efficacy and toxicity did not differ significantly between older (>70 years) and younger patients.

The treatment of patients with PS 2 is clinically challenging, the efficacy and tolerance of ICIs in such patients being a critical question. With cytotoxic chemotherapy, NSCLC patients with PS 2 have a higher rate of chemotherapy-related complications and worse prognosis [10, 11]. A higher frequency of severe clinical events leading to discontinuation of nivolumab has been reported in NSCLC patients with poor PS or CNS metastases [12]. A recent study of 175 patients with advanced NSCLC treated with nivolumab showed that PS ≥ 2 was associated with inferior overall survival (OS), PFS, and objective response rate [13]. Several clinical trials addressing the question of ICIs in such patients are in process (NCT02879617, NCT02733159). Our data suggest that nivolumab should be avoided in unselected NSCLC patients with PS 2. The use of specific biomarkers in such patients, for example PDL1 staining of tumor samples, could be an interesting strategy for selecting patients who might benefit from ICIs; this needs prospective evaluation. PD-L1 status was not available for this study because PD-L1 immunohistochemistry was not performed routinely in our hospitals at the time of the study.

In our study, patients with symptomatic CNS metastases more frequently developed severe AEs, particularly Grade 3–4 intra-cranial hypertension. Neurological manifestations generally result from perilesional edema; it remains unknown whether that edema is due to tumor, nivolumab, or both. In a small series of five patients with advanced NSCLC and asymptomatic CNS metastases treated with nivolumab as second-line treatment, one patient had a complete response, one partial response and one stable disease whereas two patients progressed in the CNS [14]. No treatment-related or CNS metastases-related Grade ≥3 adverse events were observed. Moreover, all responses occurred quickly and were durable. Similar results were also observed in a phase II trial evaluating pembrolizumab in 18 patients with advanced NSCLC and untreated and asymptomatic brain metastases [15]: six patients (33%) had durable responses in brain metastases, including four complete and two partial responses, whereas four patients had early systemic progression requiring discontinuation of protocol treatment. Pembrolizumab was also well tolerated: all neurological adverse events were Grade ≤2 and none led to treatment discontinuation. However, no data are available regarding the safety of ICIs in patients with symptomatic or larger brain metastases. Moreover, it is difficult to distinguish between progression of the original tumor and ‘pseudo-progression’ when intracranial lesions enlarge during immunotherapy treatment [16, 17].

In our study, low PFS was significantly associated with use of CS before the first cycle of nivolumab. It remains unclear whether the use of CS has a detrimental impact on the efficiency of ICIs by balancing immune responses to tumors. In patients with advanced melanoma, administration of ipilimumab and CS reportedly did not alter clinical benefit, [18] time to treatment failure, or OS compared with patients not receiving CS [19].

We found no significant differences between older and younger patients in disease-control rate and toxicity. Aging is associated with a decline in immune function called ‘immunosenescence’. This phenomenon induces alterations in the immune system, especially T and B lymphocytes, [20] which could impact the efficacy and/ or safety of ICIs. Sgambato *et al*. discussed the frailty of elderly patients and the resultant challenges in choosing the best therapeutic strategy, their focus being on the role of ICIs [21]. Benefits from nivolumab were observed in most prespecified groups in Checkmate 017 [4] and Checkmate 057, [3] except for the group aged >75 years. Those results may be attributable to the small numbers of elderly patients included in these phase III trials. In a recent meta-analysis of nine phase III randomized clinical trials of ICIs in solid tumors (including three trials of ipilimumab, four of nivolumab, one of pembrolizumab, and one of tremelimumab), efficacy was compared between younger and older patients (age cut-off 65–70 years) in 4,725 patients [22]. No significant difference in OS was observed between younger and older patients. In another study, possible predictors of hyper-progressive disease (HPD) were evaluated in 218 patients with solid tumors [23]. Patients with HPD were older than those without HPD (66 vs. 55 years, *p* = 0.007) but no significant difference was found for OS. Regarding safety, no significant difference between age groups was reported in Checkmate-017 (4). In another Food and Drug Administration (FDA) study, a subset analysis of safety of nivolumab in elderly patients with advanced cancers [24] found no differences in terms of adverse events between younger and elderly patients. In summary, our results along with previous published data suggest benefits and toxicity of ICIs are similar in older and younger patients.

In conclusion, PS 2, CS use before the first cycle of nivolumab, and symptomatic CNS metastases have an impact on the efficacy, tolerance and survival with nivolumab. Our results suggest that nivolumab is not beneficial or safe for patients with those patients. More studies are needed to evaluate the impact of immunotherapy in these categories of patients.

## Supporting information

S1 Table Treatment-related adverse events (AEs) occurring in patients with advanced NSCLC treated with nivolumab (n = 67). (DOCX)
