# Supplementary material for: Clinical factors associated with early progression and grade 3–4 toxicity in patients with advanced non-small-cell lung cancers treated with nivolumab
Source: PLoS One. 2018 Apr 23;13(4):e0195945. doi: 10.1371/journal.pone.0195945 (PMC5912777; doi:10.1371/journal.pone.0195945)
Supplement: S1 Table — (DOCX) [file pone.0195945.s001.docx]

**S1 Table. Treatment-related adverse events (AEs) occurring in patients with advanced NSCLC treated with nivolumab (n=67).**

| **Any event** | **Any grade^*^** | **Grade 3-4^*^** |
| --- | --- | --- |
| Fatigue | 40 (60) | 23 (34) |
| Anorexia | 31 (46) | 23 (34) |
| Diarrhea | 1 (1) | 0 (0) |
| Rash | 3 (4) | 1 (1) |
| Pruritus | 4 (6) | 2 (3) |
| Arthralgia | 5 (7) | 2 (3) |
| Hypothyroidism | 3 (4) | 1 (1) |
| Confusion | 9 (13) | 9 (13) |
| Pneumonitis | 2 (3) | 2 (3) |
| Nephritis | 1 (1) | 0 (0) |
| Fever | 10 (15) | 0 (0) |

*Data presented as N (%).
